# Supplementary material for: Extensive Adaptive Variation in Gene Expression within and between Closely Related Horseshoe Bats (Chiroptera, Rhinolophus) Revealed by Three Organs
Source: Animals (Basel). 2022 Dec 6;12(23):3432. doi: 10.3390/ani12233432 (PMC9741297; doi:10.3390/ani12233432)
Supplement: Supplementary file 1 [file animals-12-03432-s001.zip › supplementary-figures.docx]

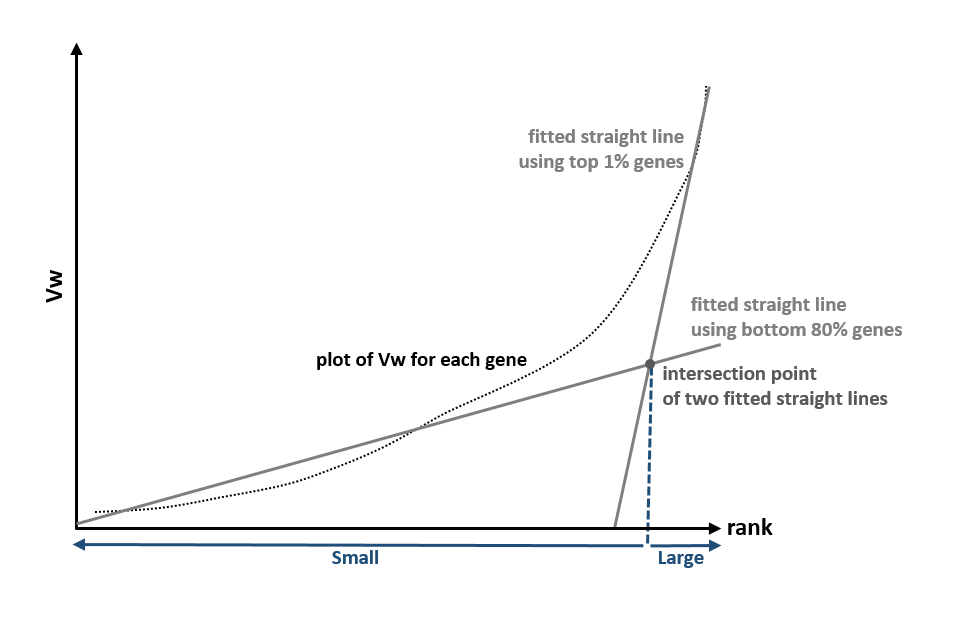


**Figure S1.** Schematic diagram defining genes with small or large expression variation among individuals within taxon using rank-based method. Gray lines are two fitted straight lines using first 1% and bottom 80% genes, respectively, and the intersection point is the cut-off point between small and large expression variation among individuals with taxon (The rank-based method is cited from Gilad et al. 2006, Blekhman et al. 2008, and Guo et al. 2016).


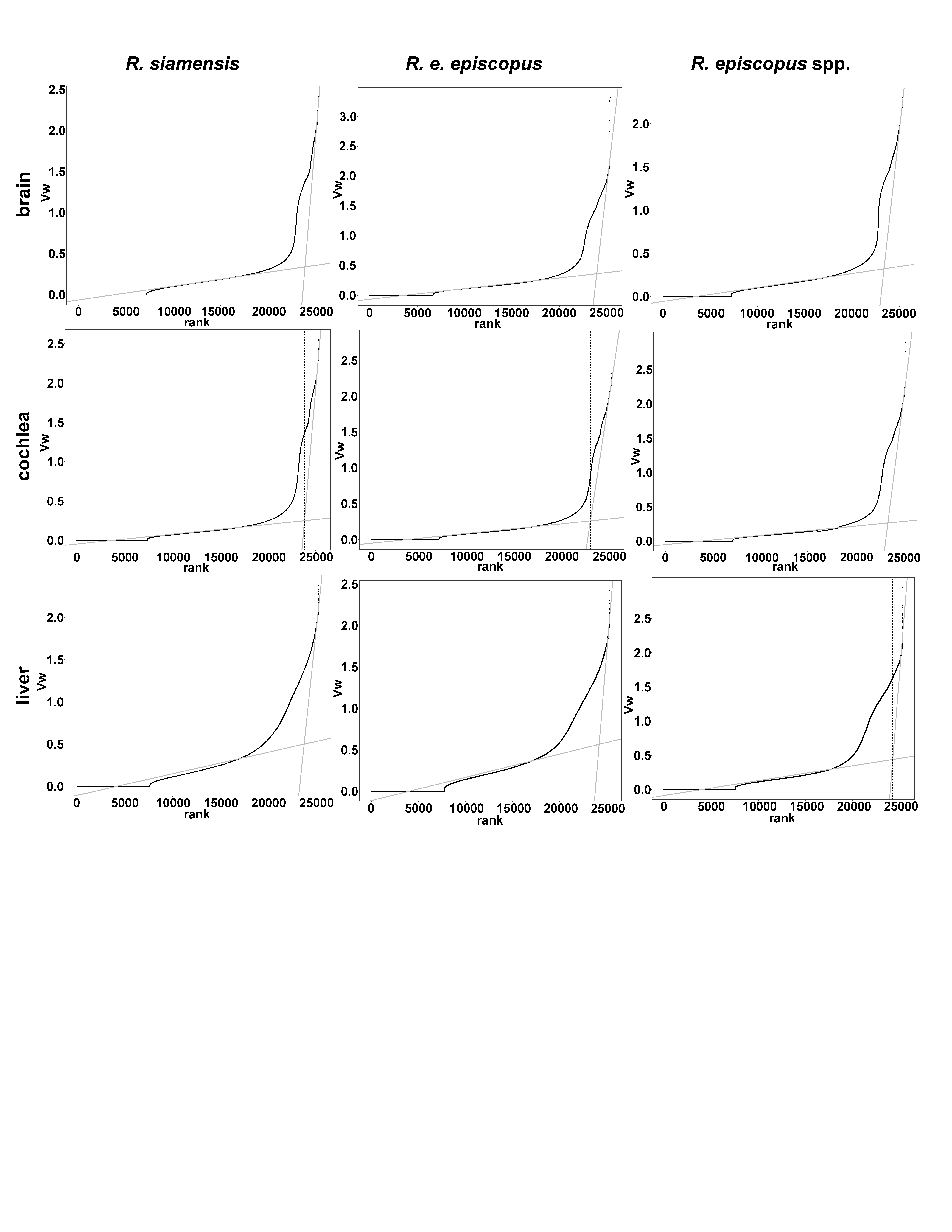


**Figure S2.** Rank distribution of expression variation among individuals within each taxon for three organs. The *X*-axis is the distribution of each gene, and the *Y*-axis is the standard deviation (SD) value of the corresponding gene in each taxon. The intersection of the two fitting lines shown in gray is the cut-off point between small and large variation among individuals within each taxon.
