# Supplementary material for: Extensive Adaptive Variation in Gene Expression within and between Closely Related Horseshoe Bats (Chiroptera, Rhinolophus) Revealed by Three Organs
Source: Animals (Basel). 2022 Dec 6;12(23):3432. doi: 10.3390/ani12233432 (PMC9741297; doi:10.3390/ani12233432)
Supplement: Supplementary file 1 [file animals-12-03432-s001.zip › supplementary-Tables S1-4.docx]

**Table S1.** Number of differentially expressed genes (DEGs) and Mann-Whitney U test (*p* value) of traits between taxa in the inter-specific and inter-subspecific comparisons. ‘*’ represents a quote from a previous study (Li et al., 2022).

| **Comparison** | **DEGs number for three organs*** | | | **Mann–Whitney U test (p-value)** | |
| --- | --- | --- | --- | --- | --- |
|  | **brain** | **cochlea** | **liver** | **RF*** | **FA** |
| Inter-specific comparison | 647 | 1069 | 692 | 0.01794 | 0.01945 |
| Inter-subspecific comparison | 528 | 608 | 1368 | 0.01066 | 0.007937 |

**Table S2.** Number of differentially expressed genes (DEGs) related to traits in the inter-specific and inter-subspecific comparisons for three organs. ‘*’ represents a quote from a previous study (Li et al., 2022).

| **Comparison** | **RF-related DEGs number*** | | | **FA-related DEGs number** | | |
| --- | --- | --- | --- | --- | --- | --- |
|  | **brain** | **cochlea** | **liver** | **brain** | **cochlea** | **liver** |
| Inter-specific comparison | 122 | 362 | 148 | 205 | 356 | 135 |
| Inter-subspecific comparison | 63 | 123 | 142 | 66 | 122 | 190 |

**Table S3.** Number of differentially expressed genes (DEGs) with large or small variation between and within taxa in the inter-specific and inter-subspecific comparisons for three organs. Vb and Vw represent the variation between taxa and among individuals within both taxa, respectively.

| **Comparison** | **Organ** | **Vb** | | **Vw** | |
| --- | --- | --- | --- | --- | --- |
|  |  | **Large variation** | **Small variation** | **Large variation** | **Small variation** |
| Inter-specific comparison | brain | 453 | 194 | 4 | 515 |
|  | cochlea | 715 | 354 | 21 | 713 |
|  | liver | 482 | 210 | 1 | 633 |
| Inter-subspecific comparison | brain | 252 | 276 | 7 | 369 |
|  | cochlea | 357 | 251 | 19 | 394 |
|  | liver | 963 | 405 | 0 | 1328 |

**Table S4.** Results of analysis of similarities (ANOSIM) (*p* value < 0.05) in the inter-specific and inter-subspecific comparisons for three organs. Vb and Vw represent the expression variation between taxa and among individuals within both taxa, respectively.

| **Comparison** | **Organ** | **Number of DEGs** | |
| --- | --- | --- | --- |
|  |  | **Vb > Vw (*p*_value < 0.05)** | **Vb < Vw** |
| Inter-specific comparison | brain | 551 | 96 |
|  | cochlea | 914 | 155 |
|  | liver | 584 | 108 |
| Inter-subspecific comparison | brain | 452 | 76 |
|  | cochlea | 551 | 57 |
|  | liver | 1068 | 300 |
