# Supplementary material for: Extensive Adaptive Variation in Gene Expression within and between Closely Related Horseshoe Bats (Chiroptera, Rhinolophus) Revealed by Three Organs
Source: Animals (Basel). 2022 Dec 6;12(23):3432. doi: 10.3390/ani12233432 (PMC9741297; doi:10.3390/ani12233432)
Supplement: Supplementary file 1 [file animals-12-03432-s001.zip › File_S1.doc]

**Methods for screening the** [**differentially expressed genes (DEGs)**](javascript:;) **whose expression variation were related to the divergence of forearm length (FA)**

Weighted gene co-expression network analysis (WGCNA) was conducted for all of the DEGs in each comparison for each organ using the R package WGCNA (version 3.6.0). The powers (soft threshold) for the combined network were set at the lowest value that optimized the scale free topology and were obtained for each comparison. A Pearson’s correlation matrix using FPKM values of DEGs was constructed and the adjacency matrix was transformed into a topological overlap matrix (TOM) to identify modules with a minimum size cut-off of 30 to generate a resulting dendrogram. The dendrogram cut height for module merging was 0.25 and the integer level of verbosity was set at 3. To identify correlations between modules and phenotypic features ([forearm length](javascript:;), *FA*), Pearson’s correlations were evaluated between *FA* and each module. The modules identified with absolute values of correlation coefficients > 0.8 and *p* < 0.01 were considered as prominent relationships to *FA*.

To further identify biologically meaningful DEGs associated with *FA* variation in each comparison for each organ, firstly, we used Pearson’s correlations to define the direct correlations between DEGs and *FA* variation (the absolute values of correlation>0.8), followed by conducting standard and network approaches to select DEGs that exhibited highly weighted correlations with *FA* in order to restrict strong correspondences between DEGs and *FA* (p <0.01). Furthermore, [a](javascript:;) [limit](javascript:;) regarding the connectedness of DEGs and modules (obtained from WGCNA) was set based on the signed eigengene-based connectivity of DEGs and [module](javascript:;) [information](javascript:;) (the absolute values of connectedness >0.9; Langfelder and Horvath 2007). The DEGs that were in the modules not related to *FA* must exhibit the absolute values of connectedness (that were obtained using this DEG and one of the modules that *FA* was significantly related to) greater than 0.8. The DEGs that met all the above restrictions concurrently were identified as DEGs related to *FA*.
