# Supplementary material for: Extensive Adaptive Variation in Gene Expression within and between Closely Related Horseshoe Bats (Chiroptera, Rhinolophus) Revealed by Three Organs
Source: Animals (Basel). 2022 Dec 6;12(23):3432. doi: 10.3390/ani12233432 (PMC9741297; doi:10.3390/ani12233432)
Supplement: Supplementary file 1 [file animals-12-03432-s001.zip › File_S2.doc]

**Code for the calculation of zFPKM, normalized FPKM values, in R (version 3.6.0)**

z_fpkm<-function(i){

if(all(i>0)) stop('Input not log2 transformed.')

if(all(!is.na(i))) stop('0\'s need to be NA\'s.')

my<-density(i,na.rm=T)$x[which.max(density(i,na.rm=T)$y)]

U<-mean(i[i>my],na.rm=T)

sigma<-(U-my)*(.5*pi)^.5

z<-(i-my)/sigma

z[z< -3]<-NA

return(z)

}
